# High-Pressure Homogenization Pre-Treatment Improved Functional Properties of Oyster Protein Isolate Hydrolysates

**DOI:** 10.3390/molecules23123344

**Published:** 2018-12-17

**Authors:** Yue Cha, Fan Wu, Henan Zou, Xiaojie Shi, Yidi Zhao, Jie Bao, Ming Du, Cuiping Yu

**Affiliations:** School of Food Science and Technology, National Engineering Research Center of Seafood, Dalian Polytechnic University, Dalian 116034, China; m18840853913@163.com (Y.C.); 15536665462@163.com (F.W.); 15589566115@163.com (H.Z.); 18309840882@163.com (X.S.); yidizhao@163.com (Y.Z.); jiebao@163.com (J.B.); mingdu121@163.com (M.D.)

**Keywords:** functional properties, shellfish proteins, high-pressure homogenization, trypsin hydrolysis

## Abstract

The effects of HPH (high-pressure homogenization) pre-treatment on the functional properties of OPIH (oyster protein isolates hydrolysates) were studied. Sodium dodecyl sulfate-polyacrylamide gel electrophoresis profiles, solubility, particle size distribution, zeta potential, surface hydrophobicity, emulsifying activity index and microstructure of emulsions were analyzed. Results indicated that HPH pre-treatment increased the accessibility of OPI to trypsin hydrolysis, resulting in decease in particle size, increase in solubility, absolute zeta potential, surface hydrophobicity and emulsifying activity index. In addition, HPH pre-treated OPIH emulsions became more uniform and the particle size of droplets decreased. These results revealed that HPH pre-treatment has the potential to modify the functional properties of OPIH.

## 1. Introduction

Proteolysis not only reduces the molecular weights of proteins, but also increases the amount of ionizable groups and exposes hydrophobic groups, which can change physical or chemical environmental interactions [1]. Hydrolysis is commonly applied to modify functional properties of native food proteins, including solubility, emulsifying and foaming properties. Trypsin is an endopeptidase; its cleavage occurs within the polypeptide chain. It was reported that hydrolysis with trypsin improved functional properties of casein and whey proteins [2], rice glutelin [3] in comparison with native proteins.

Typically, separate hydrolysis modification is relatively inefficient. It has been reported that non-thermal processing pre-treatments improved functional properties of enzymatic hydrolysates. High-pressure microfluidization pre-treatment was reported to improve emulsifying capacity of soy protein hydrolysates [4]. Microfluidization was also reported to significantly improve the hardness of the collagen hydrolysates gel [5]. Ultrasound pre-treatment can also increase emulsifying capacity of soy protein hydrolysates [6]. It has been reported that extrusion pre-treatment promoted hydrolysis of soybean protein using pancreatin and improved their emulsifying properties [7]. High-pressure homogenization (HPH) is a non-thermal food processing/preservation technology and is commonly used to process fluid in food industry. In dynamic high-pressure systems, forced-induced phenomena of cavitation, shear and turbulence and temperature rise are involved simultaneously [8]. It has been reported that conformation structure of HPH treated proteins was stretched and unfolded according to our previous studies [9,10,11]. Therefore, HPH treatment might have the potential to alter the accessibility of OPI to proteolysis and to improve functionalities of hydrolysates. However, few studies have reported this possibility.

Oysters are common and popular owing to their nutritional characteristics, special flavor and taste. Nutrient-rich oyster protein isolates (OPI) with improved functional properties are prospectively of great potential to become food additives if appropriately modified. The purpose of the present study was to explore the effects of HPH pre-treatment on hydrolysis mode and functional properties of OPI hydrolysates, and then to find eco-friendly methods to expand application of oyster protein.

## 2. Results and Discussion

### 2.1. Effects of HPH Pre-Treatment on the Enzymatic Accessibility of OPI

**Figure 1:**
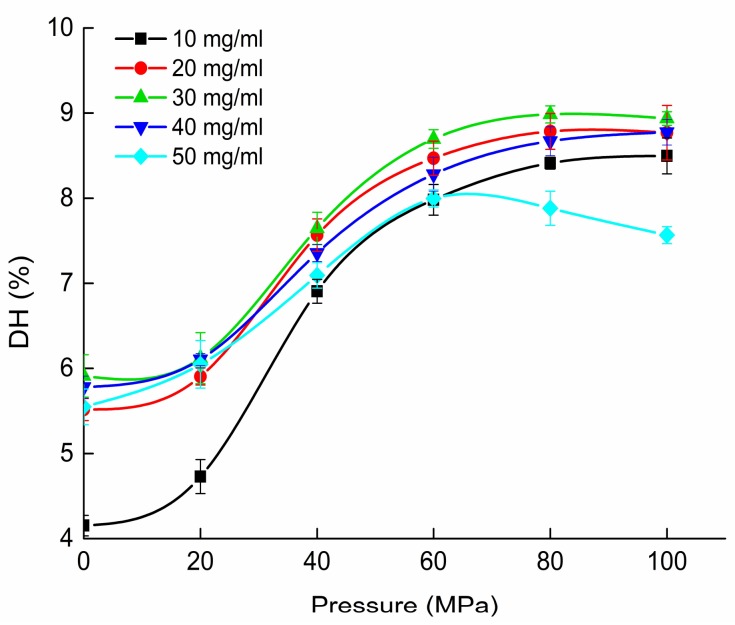
DH of HPH pre-treated hydrolysates prepared with trypsin hydrolysis under different pressures at E/S (enzyme-to-substrate ratio) = 3.0 g/100 g.

Figure 1 shows DH (degree of hydrolysis) of HPH pre-treated hydrolysates prepared with trypsin hydrolysis under different pressures. The maximum DH of native OPI was only 5.9% ± 0.3%. After HPH, DH increased significantly. As homogenization pressure increased, DH increased and reached the maximum value at concentration of 30 mg/mL under 80 MPa. At OPI concentration of 50 mg/mL, the DH of hydrolysates was lower than that of 40 mg/mL. This phenomenon can be attributed to unfolded proteins aggregating at higher concentration [12]. Based on the above results, HPH at 80 MPa with OPI concentration of 30 mg/mL was the optimal conditions to promote hydrolysis which was chosen to prepare HOPI (high-pressure homogenization pre-treated OPI) for the subsequent experiments.

**Figure 2:**
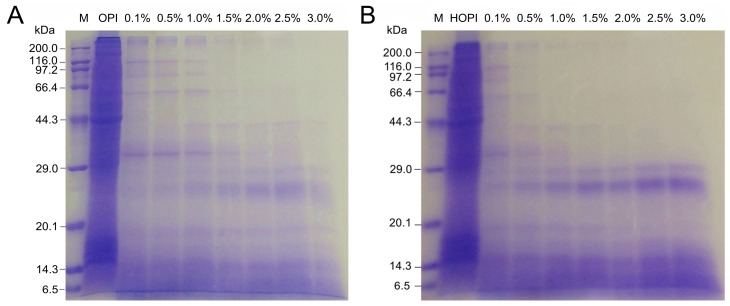
SDS-PAGE (sodium dodecyl sulfate-polyacrylamide gel electrophoresis) profiles of OPIH (**A**) and HOPIH (**B**) prepared with trypsin hydrolysis at different E/S ratios (0–3.0 g/100 g).

Protein profiles of OPIH and HOPIH (high-pressure homogenization pre-treated oyster protein isolates hydrolysates) prepared with trypsin at different E/S ratios (0–3.0 g/100 g) are shown in Figure 2A, B. As for native OPI, there were several protein bands, and the bands of 220 kDa and 42 kDa might be myosin heavy chain and actin [13,14]. There was no significant change between protein bands of OPI and HOPI. This finding was consistent with our previous study [10]. After trypsin hydrolysis, with the increase in E/S ratio, the proteins gradually degraded in both OPIH and HOPIH. Compared with OPIH, when the E/S ratio was the same, many high-molecular weight bands in HOPIH disappeared, but the number of low-molecular weight bands increased, which indicated that trypsin accessibility of OPI was better after HPH. This phenomenon might be attributed to dissociation of OPI after HPH; therefore, trypsin cleavage sites were exposed.

Table 1 shows the DH and solubility of OPIH and HOPIH prepared with different E/S ratios (0–3.0 g/100 g). As E/S ratios increased, DH of OPIH and HOPIH increased. And when E/S ratio was the same, DH of HOPIH was higher than that of OPIH, which indicated that HPH pre-treatment promoted OPI hydrolysis. This phenomenon was similar to findings in hydrolysates of soy protein isolate pretreated with high-pressure microfluidization [4]. Adequate solubility is important for functional properties of the protein. Compared with native OPI, solubility of both OPIH and HOPIH increased significantly. It has been reported that treatments of HPH and/or limited proteolysis with Alcalase significantly improved the solubility of glycinin [15]. Solubility of OPI increased from 22.4% to 39.2% after HPH pre-treatment, which was similar to HPH treated oyster proteins [10], mussel proteins [9,11], chicken protein [16] and faba bean protein [17]. Moreover, solubility gradually increased as DH increased. The decomposition of the peptide bonds induced by enzymatic hydrolysis led to the increase in ionizable amino and carboxyl groups and the decrease in molecular weights of proteins, resulting in improvements in solubility [18]. Furthermore, more hydrophilic groups were exposed after hydrolysis, and then intramolecular hydration increased [1]. When E/S ratios were the same, solubility of HOPIH was higher an OPIH. This phenomenon may be attributed to a significant increase in the accessibility of OPI after HPH pre-treatment; more OPI was easily hydrolyzed and became more soluble.

### 2.2. Effect of DH and HPH on the Particle Size Distribution and Zeta Potential of Hydrolysates

**Figure 3:**
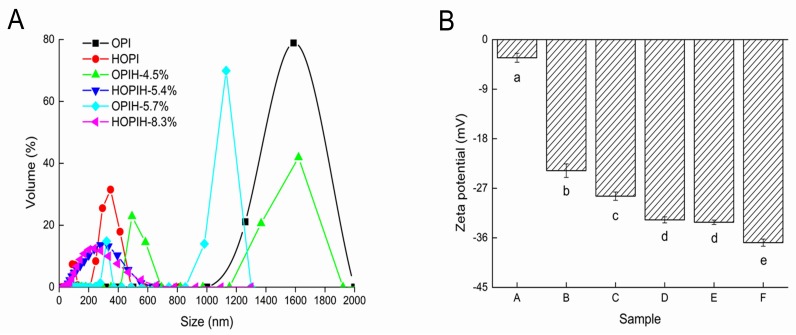
Particle size distribution (**A**) and zeta potential (**B**) of OPIH and HOPIH at different DH (a: OPI; b: HOPI; c: OPIH-4.5%; d: HOPIH-5.4%; e: OPIH-5.7%; f: HOPIH-8.3%). Different lowercase letters represent significant differences at *p* < 0.05.

Particle size distributions of OPIH and HOPIH with selected DH values are shown in Figure 3A. The particle size of native OPI showed a single peak distribution in the range of 1000–2000 nm. After HPH pre-treatment, particle size of HOPI significantly reduced to below 500 nm. This phenomenon may be due to the fact that mechanical forces generated by HPH could disrupt the structure of protein molecules, resulting in particle size reduction [19]. After trypsin hydrolysis, particle size of both OPIH and HOPIH became smaller than unhydrolyzed samples, and some particles smaller than 100 nm were detected, which might be small peptides. In addition, it was observed that the distribution of HOPIH was narrower than OPIH, suggesting that HPH pre-treatment promoted protein hydrolysis and produced more soluble small peptides. Similarly, previous studies reported that high-pressure microfluidization (120 MPa) reduced particle size of soy protein [4] and collagen [5] hydrolysates. The particle size of glycinin was reported to reduce after treatment of HPH and/or limited proteolysis with Alcalase [15].

The stability of colloidal suspension is normally determined by zeta potential analysis, which is a dependable indicator of membrane surface charge [20]. Zeta potential of OPIH and HOPIH with selected DH values is shown in Figure 3B. Compared with native OPI (3.30 ± 0.81 mV), absolute zeta potential of HOPI (23.8 ± 1.23 mV) increased significantly, which indicated that HPH resulted in more electrostatic repulsion and enhanced stability of OPI solution. This result was consistent with previous findings in oyster proteins [10], mussel proteins [9,11], chicken proteins [16,21] and hazelnut meal proteins [22] treated by HPH. The absolute potential of the hydrolyzed OPI was higher than native OPI, and the absolute potential gradually increased with the increase of DH. This phenomenon may be due to the dissociation of amino groups, resulting in changes of protein charge [23]. In addition, the absolute potential of hydrolysates pretreated by homogenization was greater than the hydrolysates without homogenization, which resulted from the promotion of hydrolysis by HPH. An increase in absolute zeta potential was also found in collagen hydrolysates after high-pressure microfluidisation (120 MPa) [5].

### 2.3. Effect of DH and HPH on H_0_ (surface hydrophobicity) of Hydrolysates

**Figure 4:**
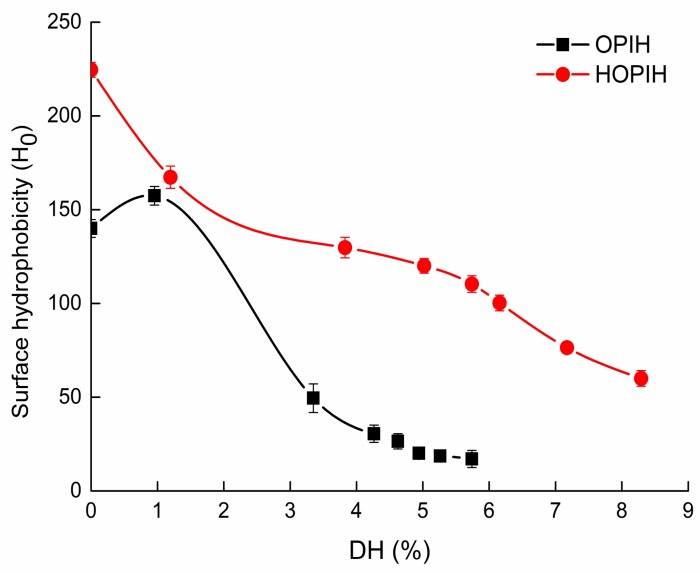
Surface hydrophobicity (*H*_0_) of OPIH and HOPIH at different DH.

Hydrophobicity plays an important role in protein conformation and functional properties [24]. As shown in Figure 4, *H*_0_ of HOPI was higher than native OPI. This result indicated that HPH could unfold OPI, leading to exposure of hydrophobic groups previously buried in protein molecules. This finding was consistent with previous studies about HPH-treated mussel proteins [9,11], oyster proteins [10] and faba bean protein [17]. *H*_0_ at 1.0% DH was greater than unhydrolyzed native OPI. This phenomenon was due to limited proteolysis resulting in the exposure of hydrophobic groups originally located inside protein molecules [25]. However, as DH increased, *H*_0_ gradually decreased, which might be due to to hydrolysis leading to a rearrangement of protein molecules, as most hydrophobic clusters got inside the aggregates [26]. As for HOPIH, *H*_0_ continued to decrease as DH increased. This result indicated that excessive hydrolysis produced shorter peptides which might have less hydrophobic binding sites than longer ones [27]. In addition, *H*_0_ of HOPIH was higher than OPIH. Similar phenomena were found in collagen hydrolysates after high-pressure microfluidisation (120 MPa) [5].

### 2.4. Effects of DH and HPH on Emulsification Properties of Hydrolysates

**Figure 5:**
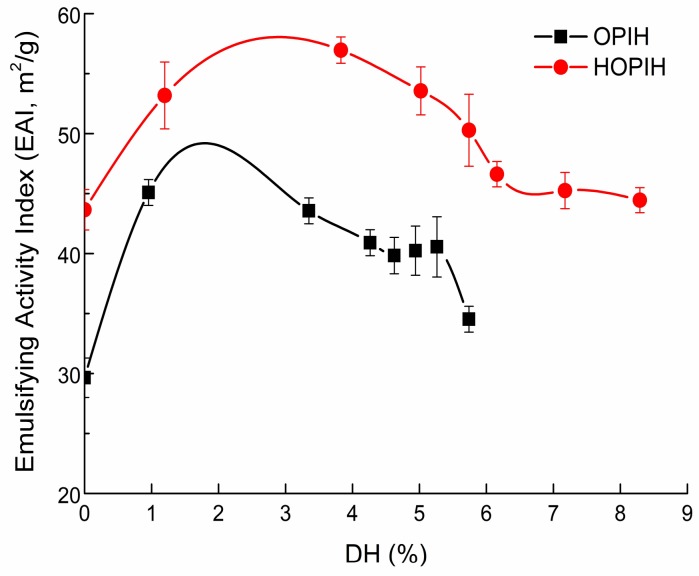
Emulsifying activity index (EAI) of OPIH and HOPIH at different DH.

As shown in Figure 5, EAI (emulsifying activity index) of HOPI was higher than OPI, indicating that EAI improved after HPH. Similar results were found in HPH modified oyster proteins [10], mussel proteins [9,11], chicken proteins [16] and peanut protein [28]. EAI of hydrolysates (OPIH and HOPIH) decreased with the increase of DH, which may be associated with the interfacial tension [29]. In addition, the decrease in EAI resulted from decreased surface hydrophobicity and protein molecular weight [30]. In addition, EAI of HOPIH was higher than OPIH, which was due to a reduction in particle size and exposure of hydrophobic groups. HOPIH was easily adsorbed at the oil-water interface, resulting in higher EAI. Similar results were found in collagen hydrolysates treated by high-pressure microfluidisation (120 MPa) [5].

**Figure 6:**
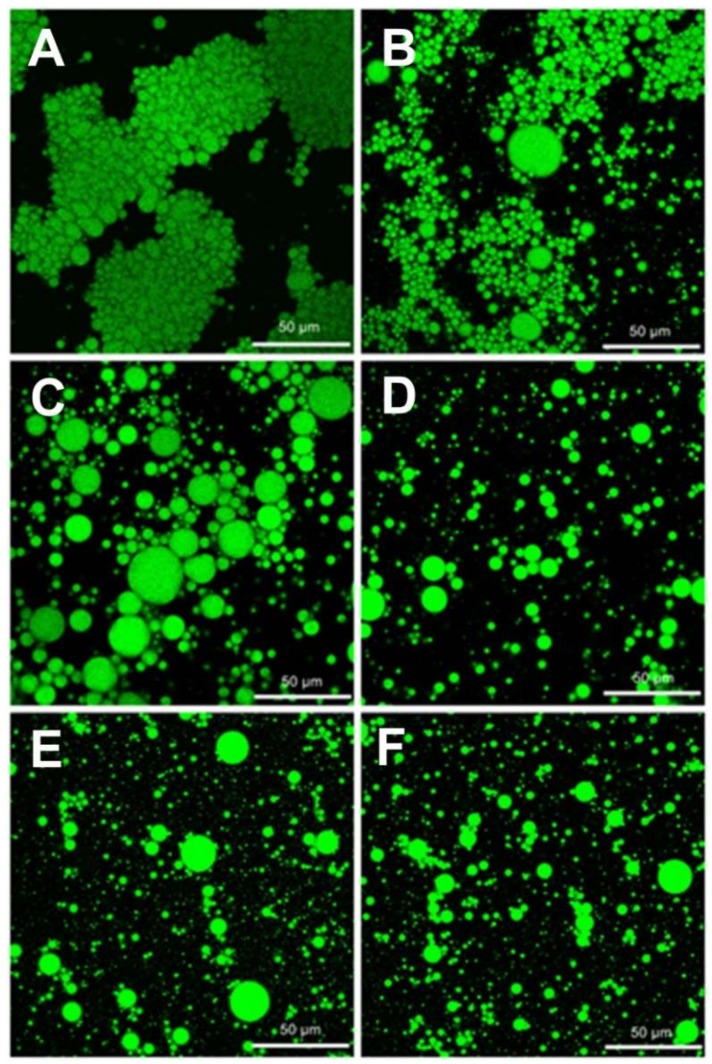
Microstructure of fresh emulsions containing 10 vol% oil and 10 mg/mL protein samples at pH 7.0 ((**A**)OPI; (**B**) HOPI; (**C**) OPIH-4.5%; (**D**) HOPIH-5.4%; (**E**) OPIH-5.7%; (**F**) HOPIH-8.3%. Scale bars = 50 μm).

Figure 6 shows the microstructures of fresh emulsions prepared from hydrolysates at different DH values. Droplets flocculation was observed in the emulsion prepared from native OPI (Figure 6A). The instability of emulsion might be due to the fact that the low solubility of native OPI was not sufficient to cover the surface of droplets, resulting in droplet cross-linking and bridging flocculation [31]. As DH increased, the particle size of droplets in OPIH decreased, and emulsions became more uniform. The results were due to the soluble proteins and peptides with higher surface activity produced; therefore, the emulsifying capability improved [32]. Compared with emulsions of OPIH, emulsions of HOPIH were more uniform and particle size of droplets was smaller, which resulted from the promotion of hydrolysis by HPH. In Figure 6E,F, there were some big droplets, which might be due to excessive proteolysis [4].

## 3. Materials and Methods

### 3.1. Materials

Oysters (*Magallana gigas*) were purchased from a local market. Trypsin (trypsin 1:250 from porcine pancreas, >250 N.F.U/mg) and ANS (1,8-Anilinonaphthalenesulfonate) was purchased from Sigma (St. Louis, MO, USA).

### 3.2. HPH Pre-Treatment of OPI

Briefly, 1000 g of minced oyster was mixed with 3.0 L of water and adjusted to pH 10.0. The dispersion was stirred for 2 h at 25 °C and centrifuged at 10,000 g for 15 min. Supernatant was then adjusted to pH 5.0, and centrifuged at 10,000 g for 20 min. Precipitates were dispersed in water at different concentrations (10–50 mg/mL) at 7.0, and then equilibrated for 30 min at 25 °C. High-pressure homogenizer (GEA Niro Soavi model Panda Plus 2000, Parma, Italy) was used to treat OPI dispersion at 20-100 MPa for 2 cycles. After homogenization, HOPI was hydrolyzed immediately.

### 3.3. Preparation of OPI and HOPI Hydrolysates (OPIH and HOPIH) at Different Degree of Hydrolysis (DH)

OPI and HOPI dispersion was trypsin-hydrolyzed at pH 8.0 and 37 °C in a water bath. E/S ratios from 0.1–3.0 g/100 g were used to reach required DH. DH was determined using pH-stat method [33]. NaOH (0.1 M) was added to maintain constant pH. PMSF (final concentration 1.0 mM) was added to stop hydrolysis [15]. DH was calculated as described previously [5].

### 3.4. Sodium Dodecyl Sulphate-Polyacrylamide Gel Electrophoresis (SDS-PAGE)

SDS-PAGE was performed as reported [34]. Briefly, samples were dispersed in SDS sample buffer to 2.0 mg/mL, boiled for 10 min, and loaded onto the gel. SDS-PAGE was conducted with a 5% acrylamide stacking gel and a 12% separating gel. After electrophoresis, Coomassie Brilliant Blue R-250 was used to stain gels.

### 3.5. Measurement of Protein Solubility

Samples were diluted in water (pH 7.0) to 30.0 mg/mL. The mixtures were stirred for 1 h at 25 °C and then centrifuged at 10,000 *g* for 10 min at 25 °C. Protein content was determined by biuret method using bovine serum albumin as standard. Solubility was expressed as the percentage of soluble protein in supernatant relative to total protein content in samples.

### 3.6. Measurement of Particle Size Distribution and Zeta Potential

Laser diffractometer (Malvern; ZETASIZER-3000HS) was used to analyze particle size and zeta potential as reported [35]. Briefly, particle size distribution and zeta potential were measured at concentration of 0.5 and 1.0 mg/mL.

### 3.7. Measurement of Surface Hydrophobicity (H_0_)

*H*_0_ was determined as reported using ANS as fluorescent probe [36]. Samples were diluted in 0.01 M phosphate buffer (pH 7.2) to 1.0–10.0 mg/mL. Then, 10.0 µL of ANS was mixed with 4.0 mL of samples. Fluorescence intensity was detected using a spectrophotometer (F-2700, Hitachi, Tokyo, Japan). *H*_0_ was expressed from the initial slope of the fluorescence intensity versus protein concentration plot of the serial dilutions.

### 3.8. Emulsification Properties

Emulsifying activity index (EAI) were detected as reported [37]. Briefly, 90.0 mL of samples (10.0 mg/mL) was mixed with 10.0 mL of soybean oil and stirred with high-speed blender (IKA, Staufen, Germany) at 20,000 rpm for 2 min. Then 50.0 µL of emulsion was mixed with 5.0 mL of SDS (1.0 mg/mL). The absorbance of diluted emulsion was read at 500 nm with spectrophotometer. EAI was expressed according to Equation (1):

(1) $$\mathrm{EAI}\;(m^{2}/g)=\frac{2\times 2.303\times A\times D}{c\times \emptyset \times \left(1-\theta \right)\times 10000}$$

where *A* is the absorbance of emulsion. *D* is dilution factor, c is concentration of samples (g/mL), ∅ is optical path, and θ is fraction of oil.

The microstructures of the emulsion prepared as described above were visualized using a Leica TCS SP8 confocal laser scanning microscope (Leica, Heidelberg, Germany) as reported [6]. Nile Red was used to stain oil phase with excitation at 488 nm.

### 3.9. Statistical Analysis

Three independent trials were carried out for each treatment. Analyses were assessed by SPSS v17.0. (IBM Corporation, Armonk, NY, USA) Data are expressed as mean ± SD. Data were subjected to one-way ANOVA followed by Duncan’s multiple range tests. *p* < 0.05 was considered as statistically significant.

## 4. Conclusions

HPH pre-treatment increased accessibility of OPI to trypsin hydrolysis, resulting in a decease in particle size, increase in solubility, absolute zeta potential, surface hydrophobicity and emulsifying activity index. In addition, HPH pre-treated OPIH emulsions became more uniform and particle size of droplets decreased. These results revealed that HPH pre-treatment has the potential to modify functional properties of OPIH.

## Figures and Tables

**Table 1:** DH and solubility of OPIH and HOPIH prepared with different E/S ratios.

E/S Ratio (g/100 g)	DH (%)		Solubility (%)	
	OPIH	HOPIH	OPIH	HOPIH
0	-	-	22.4 ± 0.5 ^a^	39.2 ± 1.2 ^b^
0.1	1.0 ± 0.1 ^a^	1.2 ± 0.1 ^b^	55.7 ± 0.7 ^c^	60.4 ± 0.8 ^e^
0.5	3.4 ± 0.1 ^c^	3.8 ± 0.1 ^d^	57.9 ± 0.2 ^d^	65.2 ± 0.6 ^g^
1.0	4.5 ± 0.2 ^e^	5.4 ± 0.2 ^i^	62.0 ± 0.2 ^f^	67.6 ± 0.3 ^h^
1.5	4.6 ± 0.1 ^f^	5.7 ± 0.1 ^j^	71.9 ± 0.6 ^i^	75.0 ± 0.7 ^k^
2.0	4.9 ± 0.2 ^g^	6.1 ± 0.1 ^k^	71.6 ± 0.4 ^i^	78.0 ± 0.2 ^m^
2.5	5.3 ± 0.1 ^h^	7.2 ± 0.1 ^l^	72.7 ± 0.7 ^j^	83.1 ± 0.9 ^n^
3.0	5.7 ± 0.1 ^j^	8.3 ± 0.1 ^m^	75.8 ± 0.2 ^l^	91.2 ± 0.1 ^o^

Different lowercase letters in the same column represent significant differences at *p* < 0.05.

## Abbreviations

HPH	high-pressure homogenization
OPI	oyster protein isolates
OPIH	oyster protein isolates hydrolysates
HOPI	high-pressure homogenization pre-treated OPI
HOPIH	high-pressure homogenization pre-treated oyster protein isolates hydrolysates
DH	degree of hydrolysis
E/S	enzyme-to-substrate ratio
SDS-PAGE	sodium dodecyl sulfate-polyacrylamide gel electrophoresis
*H*_0_	surface hydrophobicity
EAI	emulsifying activity index
ANS	1,8-Anilinonaphthalenesulfonate

## References

[1] Zhao G., Liu Y., Zhao M., Ren J., Yang B. (2011). Enzymatic hydrolysis and their effects on conformational and functional properties of peanut protein isolate. Food Chem..

[2] Chobert J.M., Bertrand-Harb C., Nicolas M.G. (1988). Solubility and emulsifying properties of caseins and whey proteins modified enzymically by trypsin. J. Agric. Food Chem..

[3] Xu X., Liu W., Liu C., Luo L., Chen J., Luo S., McClements D.J., Wu L. (2016). Effect of limited enzymatic hydrolysis on structure and emulsifying properties of rice glutelin. Food Hydrocoll..

[4] Chen L., Chen J., Yu L., Wu K. (2016). Improved emulsifying capabilities of hydrolysates of soy protein isolate pretreated with high pressure microfluidization. LWT–Food Sci. Technol..

[5] Zhang Y., Zhang Y., Liu X., Huang L., Chen Z., Cheng J. (2017). Influence of hydrolysis behaviour and microfluidisation on the functionality and structural properties of collagen hydrolysates. Food Chem..

[6] Chen L., Chen J., Ren J., Zhao M. (2011). Effects of ultrasound pretreatment on the enzymatic hydrolysis of soy protein isolates and on the emulsifying properties of hydrolysates. J. Agric. Food Chem..

[7] Chen L., Chen J., Ren J., Zhao M. (2011). Modifications of soy protein isolates using combined extrusion pre-treatment and controlled enzymatic hydrolysis for improved emulsifying properties. Food Hydrocoll..

[8] Floury J., Desrumaux A., Axelos M.A.V., Legrand J. (2003). Effect of high pressure homogenisation on methylcellulose as food emulsifier. J. Food Eng..

[9] Yu C., Cha Y., Wu F., Xu X., Qin Y., Li X., Du M. (2018). Effects of high-pressure homogenisation on structural and functional properties of mussel (*Mytilus edulis*) protein isolate. Int. J. Food Sci. Technol..

[10] Yu C., Wu F., Cha Y., Qin Y., Du M. (2018). Effects of high-pressure homogenization at different pressures on structure and functional properties of oyster protein isolates. Int. J. Food Eng..

[11] Yu C., Wu F., Cha Y., Zou H., Bao J., Xu R., Du M. (2018). Effects of high-pressure homogenization on functional properties and structure of mussel (*Mytilus edulis*) myofibrillar proteins. Int. J. Biol. Macromol..

[12] Wang X.S., Tang C.H., Li B.S., Yang X.Q., Li L., Ma C.Y. (2008). Effects of high-pressure treatment on some physicochemical and functional properties of soy protein isolates. Food Hydrocoll..

[13] Bryant M.J., Flint H.J., Sin F.Y.T. (2006). Isolation, Characterization, and Expression Analysis of Three Actin Genes in the New Zealand Black-Footed Abalone, *Haliotis iris*. Mar. Biotechnol..

[14] Suzuki M., Kobayashi Y., Hiraki Y., Nakata H., Shiomi K. (2011). Paramyosin of the disc abalone Haliotis discus discus: Identification as a new allergen and cross-reactivity with tropomyosin. Food Chem..

[15] Luo D., Zhao Q., Zhao M., Yang B., Long X., Ren J., Zhao H. (2010). Effects of limited proteolysis and high-pressure homogenisation on structural and functional characteristics of glycinin. Food Chem..

[16] Saricaoglu F.T., Gul O., Tural S., Turhan S. (2017). Potential application of high pressure homogenization (HPH) for improving functional and rheological properties of mechanically deboned chicken meat (MDCM) proteins. J. Food Eng..

[17] Yang J., Liu G., Zeng H., Chen L. (2018). Effects of high pressure homogenization on faba bean protein aggregation in relation to solubility and interfacial properties. Food Hydrocoll..

[18] Mcclements D.J., Gumus C.E. (2016). Natural emulsifiers—Biosurfactants, phospholipids, biopolymers, and colloidal particles: Molecular and physicochemical basis of functional performance. Adv. Colloid Interface Sci..

[19] Dumay E., Chevalier-Lucia D., Picart-Palmade L., Benzaria A., Gràcia-Julià A., Blayo C. (2013). Technological aspects and potential applications of (ultra) high-pressure homogenisation. Trends Food Sci. Technol..

[20] Fievet P., Szymczyk A., Labbez C., Aoubiza B., Simon C., Foissy A., Pagetti J. (2001). Determining the zeta potential of porous membranes using electrolyte conductivity inside pores. J. Colloid Interface Sci..

[21] Chen X., Xu X., Zhou G. (2016). Potential of high pressure homogenization to solubilize chicken breast myofibrillar proteins in water. Innov. Food Sci. Emerg. Technol..

[22] Saricaoglu F.T., Gul O., Besir A., Atalar I. (2018). Effect of high pressure homogenization (HPH) on functional and rheological properties of hazelnut meal proteins obtained from hazelnut oil industry by-products. J. Food Eng..

[23] Panyam D., Kilara A. (1996). Enhancing the functionality of food proteins by enzymatic modification. Trends Food Sci. Technol..

[24] Damodaran S., Parkin K.L. (2017). Fennema’s Food Chemistry.

[25] Avramenko N.A., Low N.H., Nickerson M.T. (2013). The effects of limited enzymatic hydrolysis on the physicochemical and emulsifying properties of a lentil protein isolate. Food Res. Int..

[26] Wu W., Hettiarachchy N., Qi M. (1998). Hydrophobicity, solubility, and emulsifying properties of soy protein peptides prepared by papain modification and ultrafiltration. J. Am. Oil Chem. Soc..

[27] Kato A., Komatsu K., Fujimoto K., Kobayashi K. (1985). Relationship between surface functional properties and flexibility of proteins detected by the protease susceptibility. J. Agric. Food Chem..

[28] Dong X., Zhao M., Yang B., Yang X., Shi J., Jiang Y. (2011). Effect of high-pressure homogenization on the functional property of peanut protein. J. Food Process Eng..

[29] Esther D.L.H., Manuel G., Rosell C.M. (2013). Particle size distribution of rice flour affecting the starch enzymatic hydrolysis and hydration properties. Carbohydr. Polym..

[30] Lam R.S.H., Nickerson M.T. (2013). Food proteins: A review on their emulsifying properties using a structure–function approach. Food Chem..

[31] Guzey D., Mcclements D.J. (2006). Formation, stability and properties of multilayer emulsions for application in the food industry. Adv. Colloid Interface Sci..

[32] Dickinson E. (2009). Hydrocolloids as emulsifiers and emulsion stabilizers. Food Hydrocoll..

[33] Ezquerra J.M., García-Carreño F.L., Civera R., Haard N.F. (1997). pH-stat method to predict protein digestibility in white shrimp (*Penaeus vannamei*). Aquaculture.

[34] Hsieh J.-F., Yu C.-J., Tsai T.-Y. (2012). Proteomic profiling of the coagulation of soymilk proteins induced by magnesium chloride. Food Hydrocoll..

[35] Rami M.L., Meireles M., Cabane B., Guizard C. (2009). Colloidal Stability for Concentrated Zirconia Aqueous Suspensions. J. Am. Ceram. Soc..

[36] Haskard C.A., Li-Chan E.C.Y. (1998). Hydrophobicity of bovine serum albumin and ovalbumin determined using uncharged (PRODAN) and anionic (ANS-) fluorescent probes. J. Agric. Food Chem..

[37] Karaca A.C., Low N., Nickerson M. (2011). Emulsifying properties of canola and flaxseed protein isolates produced by isoelectric precipitation and salt extraction. Food Res. Int..

